# Pro-197-Ser Mutation and Cytochrome P450-Mediated Metabolism Conferring Resistance to Flucarbazone-Sodium in *Bromus japonicus*

**DOI:** 10.3390/plants11131641

**Published:** 2022-06-21

**Authors:** Yuning Lan, Xinxin Zhou, Shenyuan Lin, Yi Cao, Shouhui Wei, Hongjuan Huang, Wenyu Li, Zhaofeng Huang

**Affiliations:** 1 State Key Laboratory for Biology of Plant Diseases and Insect Pests, Institute of Plant Protection, Chinese Academy of Agricultural Sciences, Beijing 100193, China; lan_yuning@163.com (Y.L.); caoyi2622@163.com (Y.C.); weishouhui@caas.cn (S.W.); hjhuang@ippcaas.cn (H.H.); lifreedomwy@163.com (W.L.); 2 Institute Control of Agrochemicals, Ministry of Agriculture and Rural Affairs, Beijing 100125, China; zxxisgood@163.com; 3 College of Bioscience and Biotechnology, Shenyang Agricultural University, Shengyang 110866, China; linshenyuan@163.com

**Keywords:** *Bromus japonicus* Thunb, flucarbazone-sodium, target site resistance, metabolic resistance, P450s, cross- and multiple resistance

## Abstract

In crop fields, resistance to acetolactate synthase (ALS)-inhibiting herbicides found in many troublesome weed species, including *Bromus japonicus* Thunb, is a worldwide problem. In particular, the development of herbicide resistance in *B. japonicus* is a severe threat to wheat production in China. The purpose of this research was to investigate the physiological and molecular basis of *B. japonicus* resistance to flucarbazone-sodium. Dose-response analysis demonstrated that, compared with the susceptible *B. japonicus* (S) population, the resistant (R) population exhibited a 120-fold increase in flucarbazone-sodium resistance. Nucleotide sequence alignment of the ALS gene indicated that the Pro-197-Ser mutation in ALS was associated with resistance to flucarbazone-sodium in the R population. The results of a malathion pretreatment study showed that *B. japonicus* might also have remarkable cytochrome P450 monooxygenase (P450)-mediated metabolic resistance. This is the first report of a Pro-197-Ser mutation and P450-mediated metabolism conferring resistance to flucarbazone-sodium in *B. japonicus*.

## 1. Introduction

Japanese brome (*Bromus japonicus* Thunb.), a diploid species (2*n* = 14), is an annual grass native to Eurasia that is mainly distributed in China and Japan [1]. Because this grass thrives in a variety of locations, including fields, pools, and road verges, where it competes with native plants for water and nutrients, it has become one of the most malignant weeds that heavily infest grains [2]. Wheat (*Triticum aestivum* L.) has become the world’s most widely produced cereal crop, covering more than 220 M ha and serving as the backbone of the global food security system [3]. Weeds are the leading crop pests in agriculture, causing yield losses in row crops [4]. When the density of *B. japonicus* in wheat fields is 4 plants per square meter, it can cause yield losses of 2.11% to 2.24% [5]. Thus, the grain and biological yields of wheat varieties are negatively affected by competition from *B. japonicus* [6].

At present, chemical herbicides are regarded as an efficient and cost-effective means to control weeds in cereal crops [7]. Farmers rely heavily on selective herbicides, primarily acetolactate synthase (ALS; EC 2.2.1.6) inhibitors such as flucarbazone-sodium, to control *B. japonicus* in wheat fields. Since their commercialization, ALS-inhibiting herbicides have been extensively utilized for weed management due to their broad-spectrum weed control properties and low toxicity to mammals [8,9]. There are five structurally diverse chemical classes of ALS inhibitors: imidazolinones (IMIs), pyrimidinyl benzoates (PTBs), triazolinones (SCTs), sulfonylureas (SUs) and triazolopyrimidines (TPs). All of these compounds act against the same target enzyme, ALS, which catalyzes two different reactions in the biosynthesis of leucine, isoleucine and valine (branched-chain amino acids): the condensation of two molecules of pyruvate to form acetolactate and the condensation of α-ketobutyrate and pyruvate to form acetohydroxybutyrate [10,11].

Nevertheless, herbicide resistance may occur with improper use and increasing application. Many weed species have obtained resistance to a broad variety of herbicides all over the world as a consequence of distinct herbicide selection pressure [12]. Herbicide-resistant weeds have two main resistance mechanisms: target-site resistance (TSR) and nontarget-site resistance (NTSR) [13]. TSR is characterized by target-site gene mutations or deletions, as well as gene copy number changes, and confers resistance to a specific chemical class of herbicides [13,14,15,16,17]. In 1987, *Lactuca serriola* L. was the first population reported to be resistant to ALS inhibitors; since then, 169 weed species have been discovered to be resistant to ALS worldwide [12]. A mutation target site, which is characterized by an amino acid substitution in the ALS protein (domains A-E), is the most common TSR mechanism conferring resistance to ALS inhibitors [18]. Eight confirmed sites in the five conserved domains of the ALS enzyme (Ala_122_, Pro_197_, Ala_205_, Asp_376_, Arg_377_, Trp_574_, Ser_653_ and Gly_654_) have shown resistance to ALS inhibitors in weed populations, and multiple amino acid substitutions at each site can result in resistance to ALS inhibitors [19]. NTSR, which may confer general resistance to herbicides with a variety of action mechanisms, includes changes in herbicide absorption, translocation and metabolism [13,14,17,20]. In herbicide-resistant weedy plants, only a few NTSR genes containing cytochrome P450 monooxygenases (P450s) have been found and described [21].

There have been no previous reports on the mechanisms by which *B. japonicus* has developed ALS-mediated resistance in China. Additionally, due to the strong tillering capacity of *B. japonicus* and the change in farming methods, *B. japonicus* has become the main weed in wheat fields in Hebei Province, China. Since ALS inhibitors have been applied for a long period of time, resistant *B. japonicus* populations have been discovered [22]. The purpose of this research was to (1) detect the resistance levels in different *B. japonicus* populations; (2) determine the underlying mechanism of *B. japonicus* resistance to flucarbazone-sodium; and (3) investigate cross- and multiple resistance statuses with other herbicides.

## 2. Results

### 2.1. Resistance Level Evaluation

**Figure 1:**
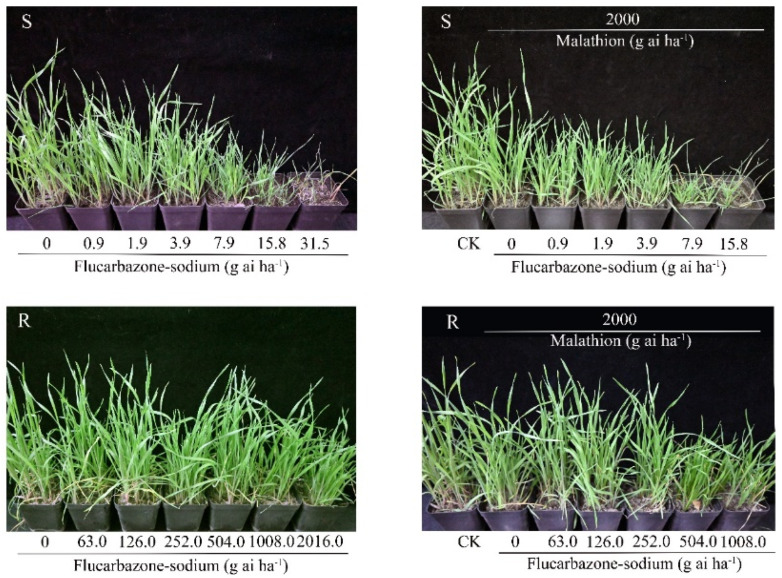
Whole-plant dose-response analysis of the resistance to flucarbazone-sodium and to flucarbazone-sodium with malathion in the S and R *B. japonicus* populations at 21 DAT.

**Figure 2:**
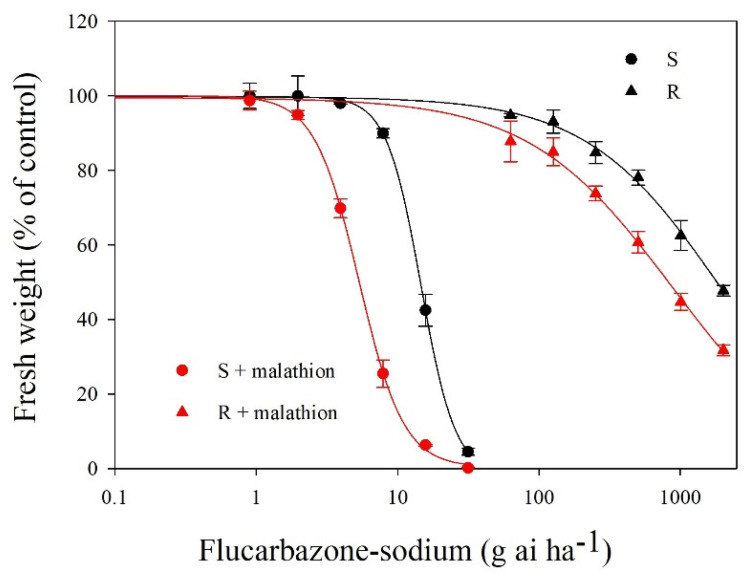
Dose-response curves of the plant growth of the *B. japonicus* populations. Vertical bars indicate the SEs (all R^2^ > 0.99).

The R and S populations showed different dose-responses to flucarbazone-sodium in whole-plant dose-response tests. As shown in Figure 1, compared with the S population, the R population was resistant to flucarbazone-sodium. The S population was unable to survive after treatment with a dose of 31.5 g ai ha^−1^ flucarbazone-sodium (the label rate). The GR_50_ values of the R and S *B. japonicus* populations were 1900.3 and 14.5 g ai ha^−1^, respectively, based on statistical assessment of the percent injury at 21 DAT for flucarbazone-sodium (Figure 2). These findings revealed that the R population was 120-fold more resistant to flucarbazone-sodium than the S population (Table 1).

### 2.2. ALS Activity Assay

**Figure 3:**
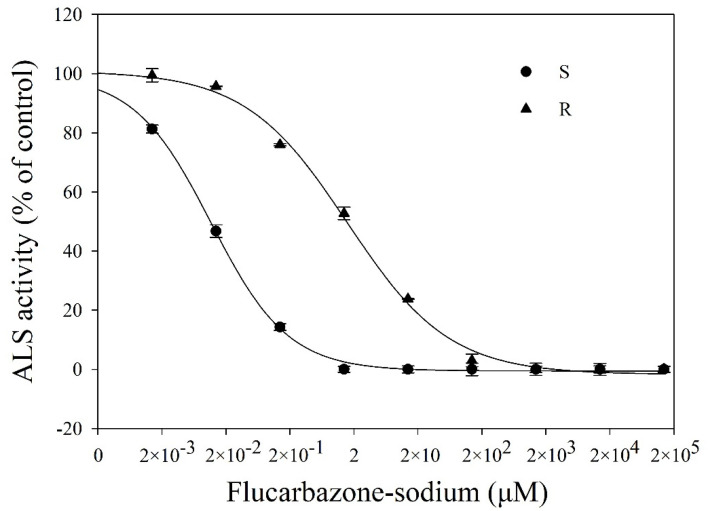
Dose-response curves of ALS activity in the S and R populations. ALS activity is expressed as a percentage of activity based on the untreated control. Vertical bars indicate the SEs (both R^2^ > 0.99).

To identify if there was variation in the sensitivity of the ALS enzyme in the two *B. japonicus* populations, ALS activity was assayed with a series of doses of flucarbazone-sodium. ALS within the R population was found to be significantly resistant to flucarbazone-sodium after its administration. The I_50_ values of the R and S populations were 1.3 × 10^−2^ and 1.9 μM, respectively, as shown in Figure 3. These results suggested that the R population was approximately 130-fold more resistant to flucarbazone-sodium than the S population (Table 1).

### 2.3. ALS Gene Sequencing

**Figure 4:**
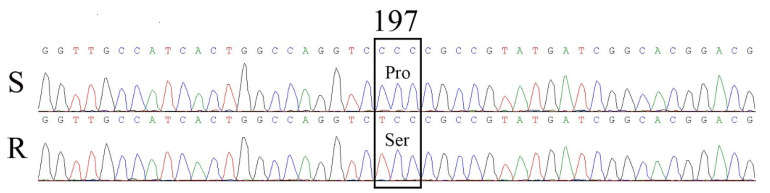
Comparison of the partial sequences of the ALS gene from the S and R populations. The amino acid position of the ALS gene was based on *Arabidopsis*.

From the R and S *B. japonicus* populations, partial *ALS* genes were sequenced. DNA sequence analyses of the *ALS* genes revealed a single nucleotide substitution of CCC to TCC at codon 197, resulting in a Pro-197-Ser mutation in the R population (Figure 4). All 10 sequenced plants containing the Pro_197_ mutation were homozygotes. Moreover, no other known amino acid mutations were identified at the Ala_122_, Ala_205_, Asp_376_, Arg_377_, Trp_574_, Ser_653_ or Gly_654_ positions in any plants sequenced in the R population.

### 2.4. Effects of Malathion Pretreatment on Metabolic Resistance

With malathion pretreatment, there was a distinct dose-response to flucarbazone-sodium in the S and R populations. R and S plants were clearly injured by malathion pretreatment with flucarbazone-sodium compared to herbicide alone. As shown in Figure 1, following the application of malathion plus flucarbazone-sodium, the fresh weights of the R and S plants declined dramatically, and the GR_50_ value was reduced by almost 60% (Table 1). These findings suggest that the P450-mediated increase in metabolism may reduce the resistance of the R population.

### 2.5. Cross- and Multiple-Herbicide Resistance Testing

To determine the resistance status of the R population, the responses of R plants administered ALS-, ACCase-, PSII- and HPPD-inhibiting herbicides at the suggested rates were investigated. Based on the surviving plants, we found that the R population was also resistant to other ALS inhibitors, such as pyroxsulam and mesosulfuron-methyl. For the ACC, PSII and HPPD inhibitors, both the R and S *B. japonicus* populations could tolerate the toxicity of these herbicides. At the recommended dose on the tag, the survival rates were more than 90% (Table 2).

## 3. Discussion

Weed resistance can be more easily developed to ALS inhibitors and ACCase inhibitors, according to Beckie and Tardif [23]. Since 2004, ALS-inhibiting herbicides have been widely used in China for weed control in wheat fields, especially for the suppression of ACCase-resistant weed species [24]. Herbicide resistance has commonly evolved as a result of the long-term usage of herbicides with a single mode of action (MOA) [25]. ALS mutations providing resistance have been found in a variety of dicotyledonous and monocotyledonous weed species. The molecular basis of the ALS mutations that confer resistance in *B. japonicus* has not been thoroughly investigated. Previous studies reported that Pro-197-Ser [18] and Ser-653-Asn [3] mutations were found in down brome (*Bromus tectorum* L.). In addition, *ALS* gene overexpression and enhanced metabolism conferring resistance to ALS inhibitors were documented in barren brome (*Bromus sterilis* L.) [26]. In this study, ALS sequencing analysis revealed that the Pro-197-Ser mutation was present in *B. japonicus.* Pro_197_ is one of the most common sites of mutation among the eight mutation sites, and this residue can be replaced with 12 other amino acids, including Ser, His, Thr, Arg, etc. In many weed species, mutation of Pro_197_ has been linked to herbicide resistance to triazolinones (SCTs), such as that found in *Rapistrum rugosum* L. [27] and *Apera spica-venti* L. [28].

Cytochrome P450 monooxygenases play an important role in herbicide metabolism [20,29]. Malathion inhibits P450s and is often used as a marker for metabolic resistance. In this study, malathion was able to reverse flucarbazone-sodium resistance in the R population. As shown in Figure 2, the GR_50_ values markedly decreased when the S and R populations were pretreated with malathion. This observation indicated that perhaps a P450-mediated enhancement in metabolism occurred in both the S and R populations. Although additional mechanisms of resistance (lower herbicide absorption or translocation) cannot be ruled out, the current findings are in line with earlier research that has shown possible P450-based resistance [30,31]. A similar phenomenon, in which malathion can reverse the resistance level, was also observed in rigid brome (*Bromus rigidus* Roth) [32]. However, more research is needed to test this hypothesis.

According to research on the worldwide phylogenetic classification of grass species, based on molecular DNA and morphological studies, *B. japonicus* and *T. aestivum* both belong to the subtribe Triticodae [33]. As a result, *B. japonicus* and *T. aestivum* may share more physiological similarities than other weeds in wheat fields. Through cross- and multiple-herbicide resistance testing, some selective herbicides used in wheat fields cannot effectively distinguish them. In the field study by Johnson et al. [6], treatment with pyroxsulam and flucarbazone-sodium obtained a similar result, in which these two kinds of ALS inhibitors were still effective. Therefore, in wheat fields, in addition to ALS inhibitors, other MOA herbicides (such as PS II and ACCase inhibitors) may not be effective for weed control. Thus, resistance is a major threat because our options for weed management will shrink.

In summary, this study reports the first case of flucarbazone-sodium resistance in *B. japonicus*. *B. japonicus* resistance to flucarbazone-sodium is conferred by the TSR mechanism (single nucleotide mutation) and NTSR mechanism (metabolic enhancement). Our results provide a [18] novel and better understanding of flucarbazone-sodium resistance in *B. japonicus* and the *Bromus* genus, which is beneficial for devising effective strategies for weed management.

## 4. Materials and Methods

### 4.1. Plant Materials and Growth Conditions

Mature seeds of *B. japonicus* plants were collected in wheat fields. After cleaning, seeds from 30 individual plants were compiled into a single sample in a paper bag and stored at 4 °C before use. First, seeds were vernalized in a 4% solution of Gibberellic acid for 24 h. Then, these seeds were sown in 7 × 5 × 10 cm plastic pots filled with commercial potting mixture. Seeds were cultivated in a greenhouse with 16 h of supplemental illumination and a temperature of 25/20 °C day/night. All pots were regularly watered, and fertilizers were applied at the same time. At the two- to three-leaf stage, all populations were treated with flucarbazone-sodium at the rate listed on the herbicide label (31.5 g ai ha^−1^). Visible estimates of *B. japonicus* viability were utilized after treatment with the herbicide [34]. One population with surviving seedlings was grown to maturity in a greenhouse. Putative resistant *B. japonicus* was referred to as the ‘R’ population (Table 3). By comparison, *B. japonicus* known to be susceptible to flucarbazone-sodium and the other herbicides tested in this study was referred to as the ‘S’ population.

### 4.2. Whole-Plant Dose–Response Tests

At the 3–4-leaf stage, *B. japonicus* weeds were sprayed with flucarbazone-sodium. The S population was sprayed with flucarbazone-sodium at 0.9, 1.9, 3.9, 7.9, 15.8 and 31.5 g ai ha^−^^1^. The R population was sprayed at 15.8, 31.5, 63.0, 126.0, 252.0, 504.0, 1008.0 and 2016.0 g ai ha^−^^1^ (the dose on the label was 31.5 g ai ha^−1^). Each dose–response experiment was randomized with two replicates per dose. The plant control rate was counted, and the aboveground biomass was harvested and weighed at 21 days after treatment (DAT).

### 4.3. ALS Activity Assay

For the ALS enzyme extraction and activity experiment, fresh plant tissue was collected at the 3–4-leaf stage. The methods for the ALS extraction, purification and activity assay were carried out according to the protocol described by Yu et al. [35]. The final concentrations of flucarbazone-sodium in the reaction mixtures were 0.002, 0.02, 0.2, 2, 20, 2 × 10^2^, 2 × 10^3^, 2 × 10^4^ and 2 × 10^5^ μM for each population. Each treatment was repeated twice with three repetitions.

### 4.4. ALS Gene Cloning and Sequencing

Fresh green leaf tissues from each plant (10 plants per population) were picked and flash-frozen for genomic DNA (gDNA) extraction. gDNA was extracted using a DNeasy Plant Mini kit (Tiangen Biotechnology Company Ltd., Beijing, China) in accordance with the manufacturer’s instructions. The forward and reverse primers (Table 4) were designed based on the DNA sequences of the *ALS* genes for *Bromus tectorum* L. (GenBank AF487459.1) and *Bromus sterilis* L. (GenBank MT113952.4).

PCR amplification was performed using 2 × PCR MasterMix II (Tiangen Biotechnology Company Ltd., Beijing, China). The conditions were as follows: initial denaturation for 3 min at 94 °C; 35 cycles of 94 °C for 30 s, 58 °C for 30 s, and 72 °C for 1 min; and 72 °C for 5 min for final extension. Then, all products were directly sequenced from both directions to minimize sequencing errors by Beijing Biomed Gene Technology Co., Ltd. (Beijing, China). The PCR sequences were analyzed for potential resistance-conferring mutations using Vector NTI (v.11.5, InforMax Inc., North Bethesda, MD, USA).

### 4.5. Effects of Malathion Pretreatment on Metabolic Resistance

Whole-plant dose-response tests were carried out to determine the GR_50_ values (herbicide dose required to reduce 50% of plant growth) of the different *B. japonicus* populations to flucarbazone-sodium in the absence and presence of malathion. Malathion, a known cytochrome P450 inhibitor, is usually considered an indicator of metabolic resistance [36]. In the following study, according to the materials and growth conditions, the R and S populations at the 3-leaf stage were treated with malathion (70%, oil dispersion) (2000 g ai ha^−1^) one hour before herbicide treatment [37]. The herbicide doses for the S and R populations were 0.9, 1.9, 3.9, 7.9, 15.8 and 31.5 g ai ha^−1^ and 63.0, 126.0, 252.0, 504.0, 1008.0 and 2016.0 g ai ha^−1^, respectively. Plants were kept in the greenhouse, and the aboveground biomass was weighed at 21 DAT.

### 4.6. Cross- and Multiple-Herbicide Resistance Testing

To evaluate the cross- and multiple herbicide resistance of *B. japonicus* to other ALS inhibitors and common herbicides used in wheat fields, the *B. japonicus* populations (R and S) were tested with these herbicides, as shown in Table 5. Herbicides were applied to the plants from each population at the three- to four-leaf stage. All herbicides were applied at three doses: 0.5×, 1× (recommended rate on the tag) and 2×, and sprayed as previously described. At 21 DAT, the efficacy of the different concentrations of herbicides on each population was evaluated by counting the control rate (fresh weight of the treated plants divided by that of the untreated plants). Each dose–response experiment was set up in a randomized complete block (blocked by population) design, with 10 replicates (pots) per treatment, which were repeated over time.

### 4.7. Statistical Analyses

A randomized full block design with three replicates for each treatment was used in the study. The dose-response trials on the whole plant and ALS activity assay were repeated twice. All of the data from the preceding trials were subjected to analysis of variance (ANOVA). Dose-response curves were created by a nonlinear log-logistic regression model using SigmaPlot software (v. 12.0, Systat Software, San Jose, CA, USA). The dose–response and ALS activity data were fitted to a nonlinear log-logistic regression model as follows:

Y=C+(D−C)/(1+((x)/G)^b)

In the above model, parameter *C* is the lower response limit, *D* is the upper response limit, *b* is the slope of the curve, *G* represents the GR_50_ or I_50_, and variable x indicates the dose. The resistance factor (RF) (resistance-to-susceptible ratio) based on the GR_50_ and I_50_ values was estimated to determine the level of resistance in the R relative to the S *B. japonicus* populations in the flucarbazone-sodium test.

## Figures and Tables

**Table 1:** GR_50_ and I_50_ values of *B. japonicus* populations treated with flucarbazone-sodium.

Population	Flucarbazone-Sodium		Flucarbazone-Sodium + Malathion		I_50_^b^ (μM)	RF
	GR_50_^a^ (g ai ha^−1^)	RF ^c^	GR_50_ (g ai ha^−1^)	RF		
S	14.5 (0.7)	-	6.5 (0.11)	-	1.3 × 10^−2^ (9.6 × 10^−3^)	-
R	1900.3 (29.9)	135.7	840.3 (22.5)	129.2	1.9 (0.4)	135.6

^a^ GR_50_, the herbicide rate causing 50% reduction in plant growth. ^b^ I_50_, the herbicide dose to inhibit 50% ALS activity. ^c^ RF, the ratio of the GR_50_ and I_50_ values of the resistant and susceptible populations. Values in parentheses are the SEs.

**Table 2:** Survival rates after cross- and multiple-herbicide resistance testing.

Herbicide	Dose ^a^ (g ai ha^−1^)	Population	
		S (%)	R (%)
Mesosulfuron-methyl	6.75	27.14 (0.16)	99.23 (0.22)
	**13.5**	21.63 (0.24)	98.71 (0.15)
	27.0	15.42 (0.18)	95.62 (0.11)
Pyroxsulam	7.50	20.39 (0.13)	97.94 (0.21)
	**15.0**	17.15 (0.31)	96.15 (0.09)
	30.0	16.06 (0.28)	95.32 (0.14)
Clodinafop-propargyl	33.8	95.84 (0.08)	95.44 (0.36)
	**67.5**	90.68 (0.14)	92.49 (0.11)
	135	85.22 (0.35)	90.21 (0.43)
Isoproturon	650	96.42 (0.27)	96.29 (0.08)
	**1300**	94.23 (0.11)	90.27 (0.14)
	2600	85.76 (0.18)	81.52 (0.32)
Cypyrafluone	90	96.20 (0.24)	97.23 (0.15)
	**180**	84.83 (0.19)	91.76 (0.22)
	360	75.18 (0.23)	74.28 (0.27)

^a^ The number in bold means the recommended field rate. Values in parentheses are the SEs.

**Table 3:** Geographical locations of the collection sites for the seeds of resistant *B. japonicus*.

Population	Location	Coordinates
R	Wangcun Town, Baoding City, Hebei Province	39°26′ N, 115°45′ E
S	Liuyuankou Town, Kaifeng City, Henan Province	34°53′ N, 114°20′ E

**Table 4:** Primers used in this research.

Primer	Sequence	Tm (°C)	Amplified Mutation Sites
ALS1f	5′-TTGATCCAGCGGAGATTGGAA-3′	58	Ala_122_, Pro_197_, Ala_205_
ALS1r	5′- CTGGGGTCTCGAGCATCTTC-3′		
ALS2f	5′-GCCGCATGATCGGTACG-3′	57	Asp_376_, Arg_377_
ALS2r	5′-CCACCACTTGGGATCATAGG-3′		
ALS3f	5′-ATGTGGGCGGCTCAGTATTA-3′	55	Trp_574_, Ser_653_, Gly_654_
ALS3r	5′-TCGATCCTGCCATCACCTTC-3′		

**Table 5:** Herbicides and their doses used in the cross- and multiple-herbicide resistance experiments.

Group ^a^	Herbicide ^b^	Doses ^c^ (g ai ha^−1^)
ALS inhibitor	Mesosulfuron-methyl 30 g L^−1^ OD	6.75 **13.5** 27.0
	Pyroxsulam 4% OD	7.50 **15.0** 30.0
ACCase inhibitor	Clodinafop-propargyl 15% OD	33.8 **67.5** 135
PS II inhibitor	Isoproturon 50% SC	650 **1300** 2600
HPPD inhibitor	Cypyrafluone 6% OD	90 **180** 360

^a^ ACCase, acetyl-CoA carboxylase; PS II, photosystem II complex; HPPD, 4-hydroxyphenylpyruvate dioxygenase. ^b^ OD, oil dispersion; SC, suspension concentrate. ^c^ The number in bold means the recommended field rate.

## Data Availability

Data are contained within the article.
